# The impact of breastfeeding on facial appearance in adolescent children

**DOI:** 10.1371/journal.pone.0310538

**Published:** 2024-09-17

**Authors:** Seppe Goovaerts, Ahmed M. El Sergani, Myoung Keun Lee, John R. Shaffer, Peter Claes, Seth M. Weinberg

**Affiliations:** 1 Department of Human Genetics, KU Leuven, Leuven, Belgium; 2 Medical Imaging Research Center, University Hospitals Leuven, Leuven, Belgium; 3 Department of Oral and Craniofacial Sciences, Center for Craniofacial and Dental Genetics, University of Pittsburgh, Pittsburgh, PA, United States of America; 4 Department of Human Genetics, University of Pittsburgh, Pittsburgh, PA, United States of America; 5 Department of Electrical Engineering, ESAT/PSI, KU Leuven, Leuven, Belgium; 6 Murdoch Children’s Research Institute, Melbourne, Victoria, Australia; Ohio State University, UNITED STATES OF AMERICA

## Abstract

Evidence that breastfeeding impacts the facial features of children is conflicting. Most studies to date have focused on dental and skeletal malocclusion. It currently remains unclear whether such effects are of sufficient magnitude to be detectable on outward facial appearance. Here, we evaluate the extent to which maternally reported breastfeeding is associated with 3D facial shape in a large adolescent cohort. After extracting 3D facial surfaces from MR scans in 2275 9- and 10-year-old children and aligning the surfaces in dense correspondence, we analyzed the effect of breastfeeding on shape as a dichotomous (no/yes) and semi-quantitative (to assess duration in months) variable using partial least squares regression. Our results showed no effect (p = 0.532) when breastfeeding was dichotomized. However, when treated as a semi-quantitative variable, breastfeeding duration was associated with statistically significant changes in shape (p = 3.61x 10^−4^). The most prominent facial changes included relative retrusion of the central midface, zygomatic arches, and orbital regions along with relative protrusion of forehead, cheek, and mandible. The net effect was that as breastfeeding duration increased, the facial profile in children became flatter (less convex). The observed effects on the face, however, were subtle and likely not conspicuous enough to be noticed by most observers. This was true even when comparing the faces of children breastfed for 19–24 months to children with no reported breastfeeding. Thus, breastfeeding does appear to have detectable effect on outward facial appearance in adolescent children, but its practical impact appears to be minimal.

## Introduction

Breastfeeding has numerous well-established benefits for both babies and mothers [1], most of which have to do with the nutritional and protective factors contained in breastmilk. Within the field of dentistry, some experts assert that breastfeeding is also beneficial for early facial growth and may even be conceptualized as a form of “early functional jaw orthopedics” [2]. The rationale for the claim is that breast suckling, as opposed to bottle feeding and non-nutritive sucking behaviors, produces forward traction forces and engages masticatory muscles that stimulate the coordinated growth of the midface and mandible [3, 4]. The mechanical forces involved in breastfeeding have been described in detail [5]. However, the evidence base supporting the link between breastfeeding and facial form is less solid.

Many studies have investigated the effects of breastfeeding on orofacial form and growth. These studies generally fall within the orthodontics literature and focus on measures of dental and/or skeletal malocclusion. The results of these studies are highly variable with some finding no relationship [6–8] and others reporting significant and clinically relevant effects. For the studies that do report an association, breastfeeding has been found to reduce the incidence of posterior crossbite and anterior open bite [9–14] and impact maxillofacial form, including palatal arch dimensions [15, 16]. Differences in study design, measurement approaches, and study samples are all factors contributing to these variable outcomes, making it difficult to draw definitive conclusions. Several reviews have tried to make sense of the discrepant findings [17–23]. Of these, two systematic reviews found no or equivocal evidence of a relationship between breastfeeding and malocclusion [18, 19]. In contrast, the two available meta-analyses found some evidence of association. Boronat-Catalá et al. [20] reported that breastfeeding is protective against class II malocclusion and posterior crossbite and that this protective effect increases as breastfeeding duration increases. Likewise, Thomaz et al. [21] reported that breast feeding for at least six months was protective against overjet, open bite, posterior crossbite and this protective effect increased when the breastfeeding extended to 12 months. Thus, the weight of evidence based on these prior studies suggests that breastfeeding likely reduces the risk of several common types of malocclusion.

A key limitation of prior studies is that very few focus on facial soft tissues and the ones that do only consider the face in 2D cephalometric profile, which can only capture a limited amount of facial geometry. Thus, it is unclear to what degree the purported effects translate to outward facial appearance, i.e., what a person sees in the mirror and presents to the world. In this retrospective study, we examine the relationship between breastfeeding during infancy and 3D facial surface shape in a large sample of US adolescent boys and girls. We modeled the effects in two ways: as a simple binary trait (no breastfeeding vs any amount of breastfeeding) and as semi-continuous variable (duration of breastfeeding in months). Based on previously published work, we predict the largest effects would involve the mandible and midface. However, the morphometric methods we employ here cover the full 3D facial surface and may allow us to uncover new and unexpected effects.

## Materials and methods

### Dataset description

For this retrospective cohort study, we used available, pre-existing data collected as part of the Adolescent Brain and Cognitive Development (ABCD) study, a large 10-year NIH-funded longitudinal study recruiting children starting at nine years of age at 21 US locations [24]. A total of 11,880 nine- and ten-year-old boys and girls have been enrolled, and a wide variety of data have been made available to the research community through the controlled-access NIMH data archive (https://nda.nih.gov/). Among the available data relevant for this study are whole-head magnetic resonance (MR) scans, anthropometric data, health and pregnancy history data (including breastfeeding history), and demographic data. These data were collected between 2016 and 2018. The authors did not have access to data that could lead to the identification of individual participants. The ABCD data used for the analyses is from Data Release 3.0 (February 2020) and was accessed on February 17, 2021.

ABCD data has been approved for broad sharing. Local institutional approval at KU Leuven (Protocol: S60568) and the University of Pittsburgh (Protocol: CR19080051) was granted for access and analysis of ABCD data housed in the NIMH data archive. The parents of children enrolled in the ABCD study provided written informed consent for themselves and their children to participate and have their data shared. In addition, enrolled children also provided their assent to demonstrate their willingness to be a participant in the study.

### Extraction and processing of 3D facial surfaces from MR scans

Whole head 3T T1-weighted MR scans were accessed through the NIMH data archive. The files were available in NIFTI format, which is standard file format used for MR brain scans. Details about the MR protocol and harmonization have been described previously [25, 26]. The complete 3D facial surface extraction and QC steps are described in our supplemental materials (**S1 Text**). Briefly, noise levels in each MR image were reduced by inter-subject non-rigid registration of 300 images from the dataset to each given target image using the Elastix (SimpleITK library [27] in Python) and computing a consensus image based on the median intensity values per voxel. After extracting the isosurface (*isosurface* in Matlab 2023a) and removal of internal structures, the 3D facial surface was extracted and remapped by non-rigidly registering a facial mesh template (*n* = 7160 vertices) to each image using the MeshMonk toolbox [28]. Imaging artefacts, mostly due to soft-tissue compression of the cheeks and chin, were detected in a vertex-wise manner based on a statistical shape model built from a set of high quality, manually curated images from the total dataset. Following QC and cleaning processes, 4930 MR-derived 3D facial surface images were available for further analysis.

### Breastfeeding variables

The mothers of enrolled children were asked to provide information about their breastfeeding habits. First, mothers were asked “When the child was a baby, did (you/ the biological mother) breastfeed him/her?” Responses were available for 6092 children, of which 1319 answered “No, Formula Only” and 4773 answered “Yes, s/he was breastfed.” Mothers who responded “yes” were then asked about the duration of breastfeeding. Responses were coded as 1 = several days (n = 99), 2 = 1–3 months (n = 815), 3 = 4–6 months (n = 1026), 4 = 7–9 months (n = 600), 5 = 10–12 months (n = 943), 6 = 13–18 months (n = 708), 7 = 19–24 months (n = 301), and 8 = more than 24 months (n = 236). An additional 45 mothers who answered “yes” to the initial question either did not know or did not provide a response to the follow up question on duration.

### Final study sample

The only exclusion criteria applied to this study were having an unusable 3D facial surface model and failure to provide breastfeeding data. Among the 4930 children with useable 3D facial surfaces, a total of 2275 unrelated children also had valid breastfeeding data available; this was final sample we used for our analyses. Effects were modeled as a simple binary trait and as a quantitative, semi-continuous variable to investigate duration of breastfeeding. For the initial binary response, the final sample breakdown was 434 (“no, formula only) and 1841 (yes, s/he was breastfed”). For the breastfeeding duration variable, the first category (“several days”) and the last category (“more than 24 months”) were dropped due to small sample size, leaving 2,148 children available for analysis. The final sample sizes for the duration analysis were as follows: 0 months (n = 434), 1–3 months (n = 293), 4–6 months (n = 384), 7–9 months (n = 222), 10–12 months (n = 375), 13–18 months (n = 307), and 19–24 months (n = 133). See **S1 Table** for additional details.

### Morphometric analyses

Remapped 3D facial surfaces were Procrustes aligned and adjusted for age, sex, height, weight, BMI, head size, facial size, genetic ancestry, MRI scanner, MRI software, and MRI pulse sequence parameters. Genetic ancestry was based on principal components derived previously using genome-wide data available for the ABCD cohort [29]. We used a regression-based approach to adjust for covariates; this involved predicting the facial surface from all covariates collectively, followed by subtraction of the predictions from the original coordinates, and adding the obtained residuals to the average facial surface. Because of the high noise levels in the data, we applied principal components analysis (PCA) to the adjusted shape residuals and omitted the higher order components which are known to capture imaging-related noise and idiosyncratic artefacts. Specifically, parallel analysis was used to determine which components had eigenvalues indistinguishable from those of simulated noise, leaving 60 components as a multivariate description of facial shape.

The association between breastfeeding, as a binary or semi-quantitative variable (encoded as the middle value of each duration category), and facial shape (modeled as PCs) was tested using partial least squares regression (PLSR; *plsregress* in Matlab 2023a). PSLR combines the dimensionality reduction benefits of PCA with multiple regression and is useful for uncovering the relationship between a set of reduced latent variables (like those in multivariate shape data) and an independent variable, set of variables, or another set of reduced latent variables [30]. The latent facial phenotype associated with the breastfeeding variable was obtained from the fitted PLSR shape coefficients. Significance of the effect was determined based on an empirical distribution of R^2^ obtained by testing 10^7^ permutations of the breastfeeding variable. For completeness, PLSR analysis was also repeated without the intermediate PCA step (i.e., directly on the shape coordinates) to confirm that the inclusion of this step did not affect our results or conclusions (**S1 Fig**).

## Results

**Fig 1:**
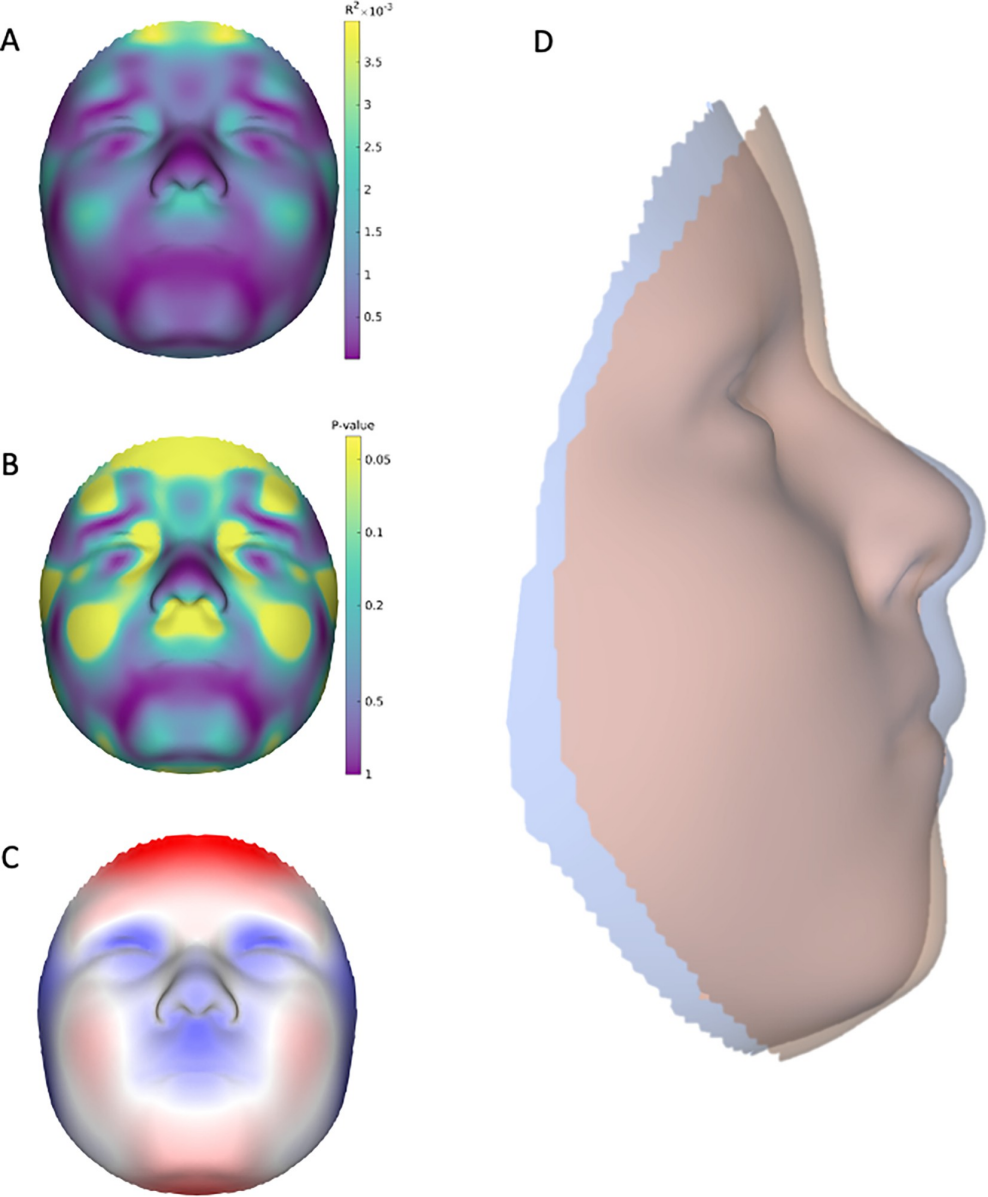
The effect of breastfeeding modeled as a semi-quantitative variable on 3D facial surface shape via PLSR. A) Heat map showing the effect of breastfeeding on the face expressed as R^2^ values; higher R^2^ values (toward the yellow) show areas where breastfeeding has a larger effect on face shape. B) Heat map showing the permutation-based p-values associated with R^2^. C) Heat map showing the direction and magnitude of the effect of breastfeeding on the face as a shift in surface normals; red shows the face moving outward, blue shows the face moving inward, and silver shows no effect. D) The effect of breastfeeding modeled as a 3D shape deformation resulting two hypothetical morphs which are then superimposed. The blue morph represents the shape in the direction of less breastfeeding, while the pink morph represents that shape in the direction of more breastfeeding. For visualization purposes the effects have been magnified 100x.

**Fig 2:**
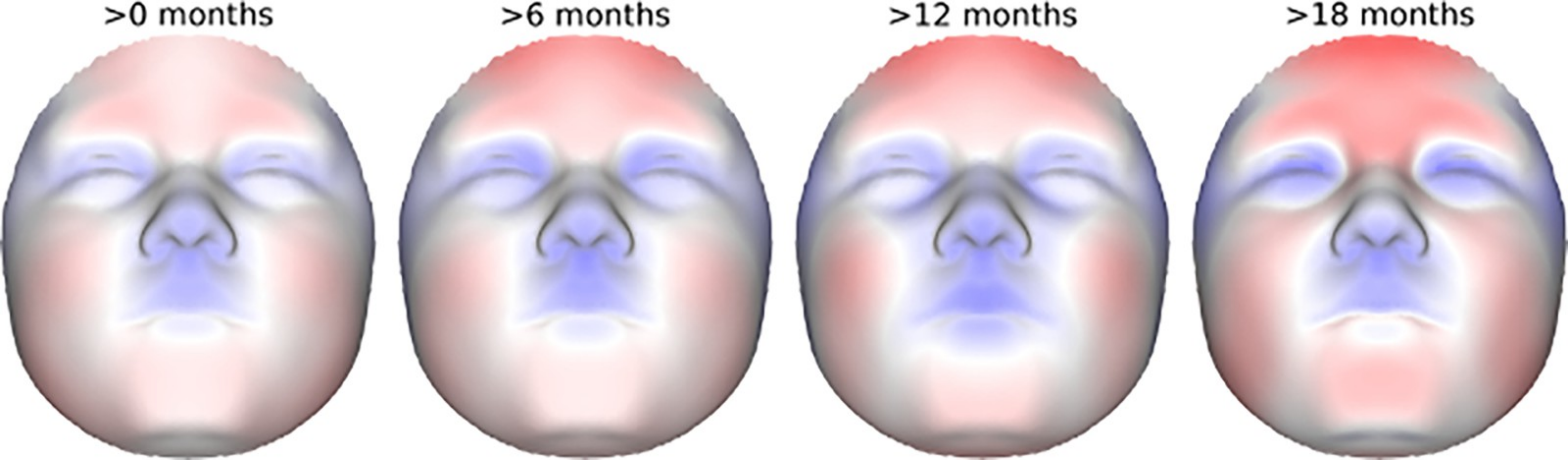
The effect of breastfeeding duration on face shape. Heat maps showing the effect of breastfeeding duration at four different thresholds on facial surface shape. Each face shows the 3D surface normal displacement at a given threshold relative to the observed mean face from the non-breastfed group. For example, the >6 month comparison shows the facial effect of breastfeeding for at least 7 months relative to the non-breastfed group. For interpretation, blue indicates that the breastfed group shows inward retrusion relative to the non-breastfed group, while red indicates outward protrusion. The colormap is on the same scale for every comparison.

We observed no significant difference in facial shape when treating breastfeeding as a simple binary yes/no variable (PSLR R^2^ = 4.29x10^-4^; p = 0.532). In contrast, when treated as a (semi) quantitative variable, breastfeeding duration in months was associated with changes in shape (PSLR R^2^ = 8.05x10^-4^; p = 3.61x 10^−4^). Looking at the R^2^ and permutation p-value facial heat maps (**Fig 1A** and **1B**, respectively) the facial regions with the strongest effects included the philtrum, cheeks, zygomatic arch, inner (medial) canthal regions of the eye, and the forehead. The magnitude and direction of effect is discernable in **Fig 1C**, which shows that increased breastfeeding duration is associated with central midface, zygomatic arch, and orbital retrusion along with concomitant forehead, cheek, and mandibular protrusion. The mandibular effect, however, was not significant. The overall impression is that increased breastfeeding is associated with a less convex facial profile. This tendency can be seen clearly when we exaggerate the effect on the face in the direction of greater or lesser breastfeeding duration (magnified +/- 100x) and then superimpose the resulting warped 3D facial surfaces (**Fig 1D**). The effect of breastfeeding duration is revealed further in **Fig 2**, which shows the progressively increasing facial shape changes at different duration thresholds.

**Fig 3:**
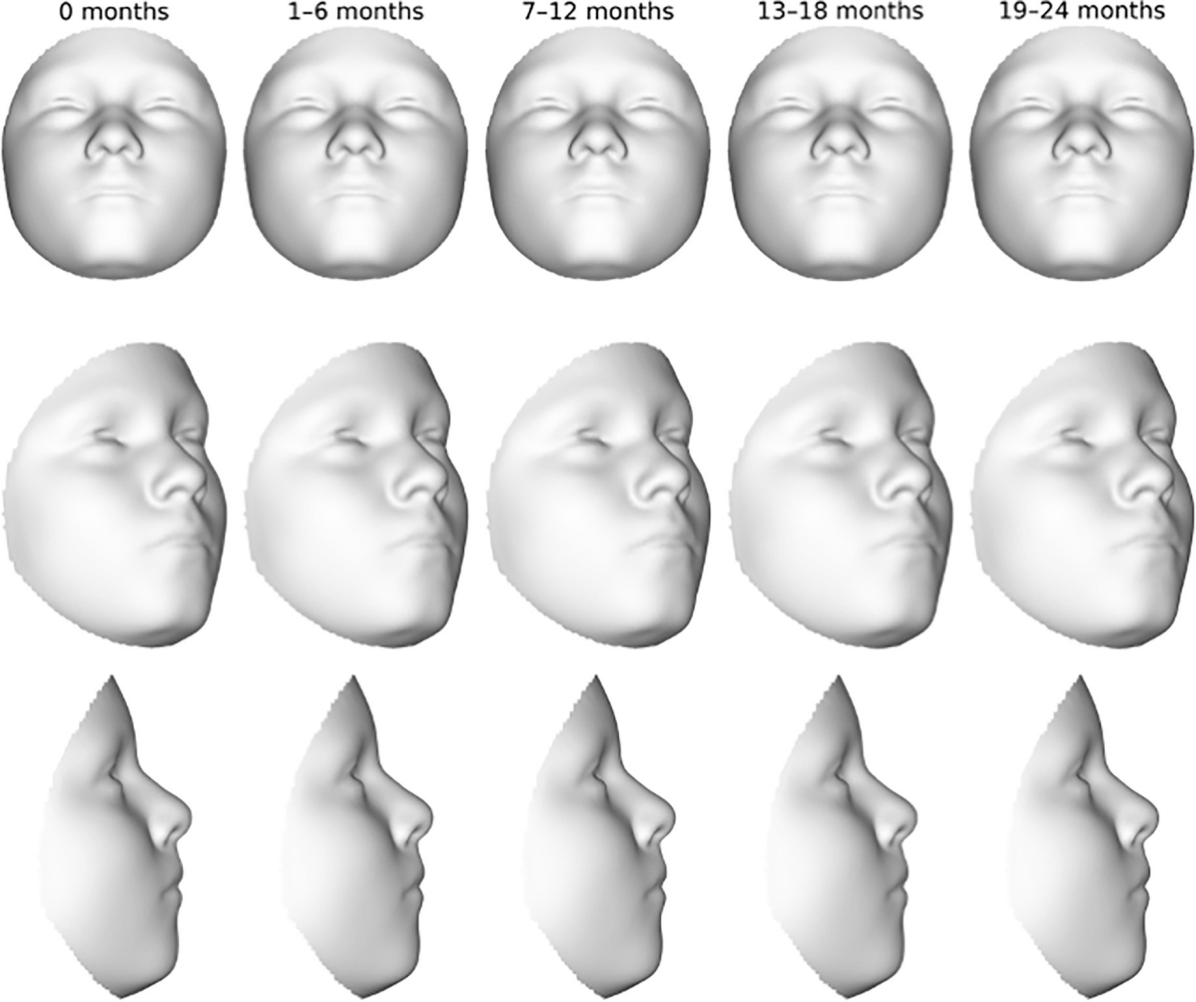
Expected changes in facial shape due to breastfeeding based on our PSLR model. *This figure shows the expected face shape as predicted from our regression model as a 3D deformation at 6-month intervals*. *Note that even by 19–24 months*, *the effects on the face are not conspicuous*.

All of the aforementioned shape changes were very subtle. In contrast to the exaggerated morphs in **Fig 1D**, the magnitude of the shape changes was very small when considered within the observed 24-month exposure timeframe. This can be appreciated in **Fig 3**, which shows the expected face shape from our regression model as a 3D deformation at 6-month intervals. At this scale, differences in facial appearance were not conspicuous, even when children were breastfed for 19–24 months.

## Discussion

We observed a statistically significant association between breastfeeding duration (measured to 24 months) and facial shape. As breastfeeding duration increased, we observed a pattern of retrusion in involving the central midface (most prominent in the philtrum and medial canthal region) and protrusion in the forehead and cheeks. Although there was a tendency toward mandibular protrusion, the effect in this region was not statistically significant. Thus, our initial prediction that breastfeeding would impact the midface and mandible was partially borne out. The combined effect of the aforementioned shape changes was one of decreased facial convexity with increased breastfeeding duration. In contrast, we found no shape differences when we simply dichotomized breastfeeding as a yes/no variable. This could be due to the fact that a large portion of our sample was breastfed for a short duration, which when combined with longer duration individuals into a single group, may have muted the impact of breastfeeding on facial shape.

It is difficult to compare our results to the prior literature because we applied an entirely different morphometric approach and focused exclusively on effects involving the facial soft-tissue surface. We have no ability to assess the underlying bones or teeth. As a result, we cannot evaluate the effects of breastfeeding on occlusion directly; only the extent to which breastfeeding impacts outward facial appearance. With that said, our finding that increased breastfeeding was associated with a suite of changes consistent with decreased facial convexity (relative midfacial retrusion) seems to accord with studies showing less risk of a class II skeletal relationship [20, 31] and reduced palate length [15]. However, given how subtleness of the facial surface changes we observed, our results are potentially also in alignment with studies reporting little or no effect of breastfeeding [6–8].

It is important to note that the morphometric approach and large sample size we used is capable of detecting very subtle shape differences. The real-world impact of these statistical effects may be negligible for all practical purposes. Indeed, our results indicate that breastfeeding had such a small effect on 3D facial surface morphology, that the predicted facial differences between an individual never breastfed and an individual breastfed for 18–24 months was barely noticeable (**Fig 3**). These results could be interpreted as challenging the conventional wisdom in orthodontics that breastfeeding is an important factor in determining positive facial growth outcomes. However, it must be kept in mind that we are looking at a different set of traits here than in the orthodontics literature. It may be that the types of occlusal changes reported to result from lack of breastfeeding minimally impact outward facial appearance. It is also possible that the effects of breastfeeding on facial surface morphology are more prominent during early childhood. Our sample was composed exclusively of 9- and 10-year-old children, so we are unable to test this hypothesis. What we can say is that by adolescence, the effect on outward facial appearance appears to be negligible. Moreover, the effects of breastfeeding could disappear altogether as the children in this study approach puberty and adulthood.

It is important to appreciate that many factors impact facial morphology, including genetic variation, diet and behavior, trauma and disease, environmental exposures throughout the lifespan, and demographic factors such as age, sex and ancestry [32, 33]. For example, common genetic variants at hundreds of chromosomal regions have now been associated with facial shape, including some with effects on the same facial features impacted by breastfeeding [34]. Together, these genetic and environmental factors collectively form the backdrop against which the effects of breastfeeding on facial morphology must be considered. While some factors (like age, sex and ancestry) can usually be adjusted for in the analysis of facial shape, it is difficult or impossible to account for all sources of variation. As a result, a complete understanding of how breastfeeding influences facial shape is currently not possible.

There are several additional limitations and caveats that must be kept in mind when interpreting these results. First, as a retrospective study using an existing data resource, we were limited to the kinds of information collected from participants. There were no additional follow-up questions about breastfeeding or about other sucking or oral habits. Therefore, we were unable to consider the full range of relevant oral behaviors in study participants. Most importantly, questions about non-nutritive sucking behaviors were not available. This is potentially relevant because such behaviors have been consistently associated with malocclusions [35] and may be more prevalent in cases of early weening [36]. This line of reasoning suggests that it may not be the lack of breastfeeding itself that leads to negative orofacial growth outcomes, but rather the suite of oral behaviors and habits that tend to replace the breastfeeding [31, 37, 38].

Another potentially important caveat in this and other studies of breastfeeding relates to the introduction of noise and/or bias into the data due to maternal reporting. Mothers may not be able to accurately recall the duration of their breastfeeding. There may also be a tendency for mothers to overestimate or mislead about their breastfeeding behaviors due to social stigma associated with not breastfeeding [39].

In summary, we can tentatively conclude that the amount of breastfeeding reported by mothers seems to have a detectable, but very small, effect on outward facial appearance in their children at 9 and 10 years of age. A prospective 3D facial surface study focused breastfeeding and other oral behaviors would help resolve some of the limitations and questions posed by these results, particularly if data on occlusion could be collected at the same time.

## Supporting information

S1 Table Demographic breakdown of final sample used in this analysis. (DOCX)

S1 Text Facial surface extraction and quality control filtering. (DOCX)

S1 Fig The effect of PCA and removal of higher order components on semi-quantitative analysis outcomes. The top row shows the results as presented throughout the manuscript. To obtain these results, PCA was performed on facial shape and higher-order, noisy components were omitted. The bottom row shows the same semi-quantitative analyses without the intermediate PCA step, i.e., performed directly on the landmarks. In the first and last column, blue indicates that the breastfed group shows inward retrusion relative to the non-breastfed group, while red indicates outward protrusion. The vertex-wise variance explained by the semi-quantitative breastfeeding variable was estimated through PLSR and a corresponding P-value was obtained empirically through permutation testing.
(TIFF)
