# Supplementary material for: The impact of breastfeeding on facial appearance in adolescent children
Source: PLoS One. 2024 Sep 17;19(9):e0310538. doi: 10.1371/journal.pone.0310538 (PMC11407646; doi:10.1371/journal.pone.0310538)
Supplement: S1 Table — (DOCX) [file pone.0310538.s001.docx]

**Table S1. Demographic breakdown of final sample used in this analysis.**

| Breastfeeding duration | Sample size | Sex | Ancestry |
| --- | --- | --- | --- |
| 0 months | N=434 | Male (N=223)  Female (N=211) | White (N = 273)  Black (N = 115)  Asian (N = 0)  NHOPI (N = 0)  AIAN (N = 7)  Other (N = 12)  Multiple (N = 23)  Unknown (N = 4) |
| 1-3 months | N=293 | Male (N=163)  Female (N=130) | White (N = 238)  Black (N = 24)  Asian (N = 0)  NHOPI (N = 0)  AIAN (N = 1)  Other (N = 13)  Multiple (N = 13)  Unknown (N = 4) |
| 4-6 months | N=384 | Male (N=198)  Female (N=186) | White (N = 319)  Black (N = 36)  Asian (N = 0)  NHOPI (N = 0)  AIAN (N = 0)  Other (N = 7)  Multiple (N = 19)  Unknown (N = 2) |
| 7-9 months | N=222 | Male (N=98)  Female (N=124) | White (N = 194)  Black (N = 15)  Asian (N = 0)  NHOPI (N = 0)  AIAN (N = 0)  Other (N = 7)  Multiple (N = 6)  Unknown (N = 0) |
| 10-12 months | N=375 | Male (N=201)  Female (N=174) | White (N = 344)  Black (N = 11)  Asian (N = 0)  NHOPI (N = 0)  AIAN (N = 1)  Other (N = 4)  Multiple (N = 12)  Unknown (N = 3) |
| 13-18 months | N=307 | Male (N=156)  Female (N=151) | White (N = 281)  Black (N = 10)  Asian (N = 0)  NHOPI (N = 0)  AIAN (N = 1)  Other (N = 5)  Multiple (N = 9)  Unknown (N = 1) |
| 19-24 months | N=133 | Male (N=69)  Female (N=64) | White (N = 114)  Black (N = 3)  Asian (N = 0)  NHOPI (N = 0)  AIAN (N = 1)  Other (N = 6)  Multiple (N = 7)  Unknown (N = 2) |

Sex and ancestry (NIH categories) self-reported by parents. NHOPI = Native Hawaiian or Other Pacific Islander. AIAN = American Indian or Alaska Native
