# Supplementary material for: The impact of breastfeeding on facial appearance in adolescent children
Source: PLoS One. 2024 Sep 17;19(9):e0310538. doi: 10.1371/journal.pone.0310538 (PMC11407646; doi:10.1371/journal.pone.0310538)
Supplement: S1 Text — (DOCX) [file pone.0310538.s002.docx]

**Text S1. Facial surface extraction and quality control filtering.**

Many of the facial surfaces extracted from MRI suffered from noise and imaging artefacts due to the MR bias field, subject fixation, and partial volume effect. In fact, almost all facial surfaces were affected by some degree of soft tissue compression of the cheeks or chin because of subject fixation. This made manual selection of high-quality images non-trivial and highly subjective. Hence, we developed a data-driven quality control and sample selection pipeline to identify high-quality images more robustly.

From the noise-reduced T1-w MRI images, the iso-surface was extracted (Matlab 2023a, *isosurface*) and internal structures were removed based on a vertex’ distance to the centroid of the whole mesh. This latter step was optional but was put in place to alleviate the chance of surface registration errors later. From the iso-surfaces, faces were extracted as dense mesh structures in correspondence (*n* = 7160 vertices) using Meshmonk [1], which involved rigid and non-rigid surface registration using a facial mesh template. From these facial surfaces, imaging artefacts were detected in a vertex-wise manner. This was done based on a statistical shape model built from a set of high quality, manually curated images (*n* = 4355) from the dataset. However, for such a reference model to effectively detect soft-tissue compression, it is key to reduce any soft-tissue compression as much as possible from the images on which the model was constructed. Therefore, as a first step, we calculated the average facial surface for each MRI scanner (*n* = 30) and built a statistical shape model using principal component analysis (PCA) to model typical machine-related shape variation, e.g., soft-tissue compression. Then, from each of the 4355 initially selected, high quality images, we subtracted the shape variation that could be explained by the set of PCs that explained 96% of variation across the MRI averages. This process visually reduced the subtle soft-tissue compression that was still present in these images. Next, a reference model was built from these ‘clean’ images using PCA and was subsequently used to detect imaging artefacts, e.g., soft tissue compression, in a vertex-wise manner. Specifically, for each of 7160 facial vertices, we constructed a distribution of 3D Euclidean distances between its position on the average face and each of the 4355 reference faces. Each vertex on a new face was then tested against this reference model, yielding a *Z*-score for each vertex that was then transformed into an outlier probability using a sigmoid activation function. A leave one out approach was used to assess the high-quality images that were used to build the statistical shape model. The resulting outlier maps demonstrate how this approach could detect various types of imaging-related artefacts ranging in severity in an automated and scalable way.

Sample selection was done based on the resulting outlier maps using a K-means clustering approach. First, the set of 500 cleanest shapes was selected based on the total number of vertices with an outlier probability less than 0.05. Since outlier probabilities were strongly correlated across vertices, we reduced the dimensionality of the outlier maps using PCA and keeping only components explaining more than 1% variance. Following, K-means++ clustering (the *K*-number is based on the variance ratio criterion) on the dimensionality-reduced outlier maps, all images in clusters devoid of top-500 shapes were flagged. The clustering was repeated for 1000 rounds to ensure robust results, and all images that were flagged ≥ 5% of the rounds were omitted.

To increase the resolution at which artefacts could be detected, we divided the face into smaller hierarchical modules using hierarchical spectral clustering based on Escoufier’s RV coefficient [2] similarly to previous works [3,4]. For each of the resulting facial modules (*n* = 31), we repeated the K-means++ procedure as above. Finally, only faces that passed the sample selection procedure in each of the 31 modules was selected for further analysis (*n* = 4930).

## **References**

1. White, J. D. et al. MeshMonk: Open-source large-scale intensive 3D phenotyping. Sci. Rep. 9, 6085 (2019).

2. Robert, P. & Escoufier, Y. A Unifying Tool for Linear Multivariate Statistical Methods: The RV-Coefficient. J. R. Stat. Soc. Ser. C Appl. Stat. 25, 257–265 (1976).

3. Claes, P. et al. Genome-wide mapping of global-to-local genetic effects on human facial shape. Nat. Genet. 50, 414–423 (2018).

4. White, J. D. et al. Insights into the genetic architecture of the human face. Nat. Genet. 53, 45–53 (2021).
