# Supplementary figures and images for: The impact of breastfeeding on facial appearance in adolescent children
Source: PLoS One. 2024 Sep 17;19(9):e0310538. doi: 10.1371/journal.pone.0310538 (PMC11407646; doi:10.1371/journal.pone.0310538)

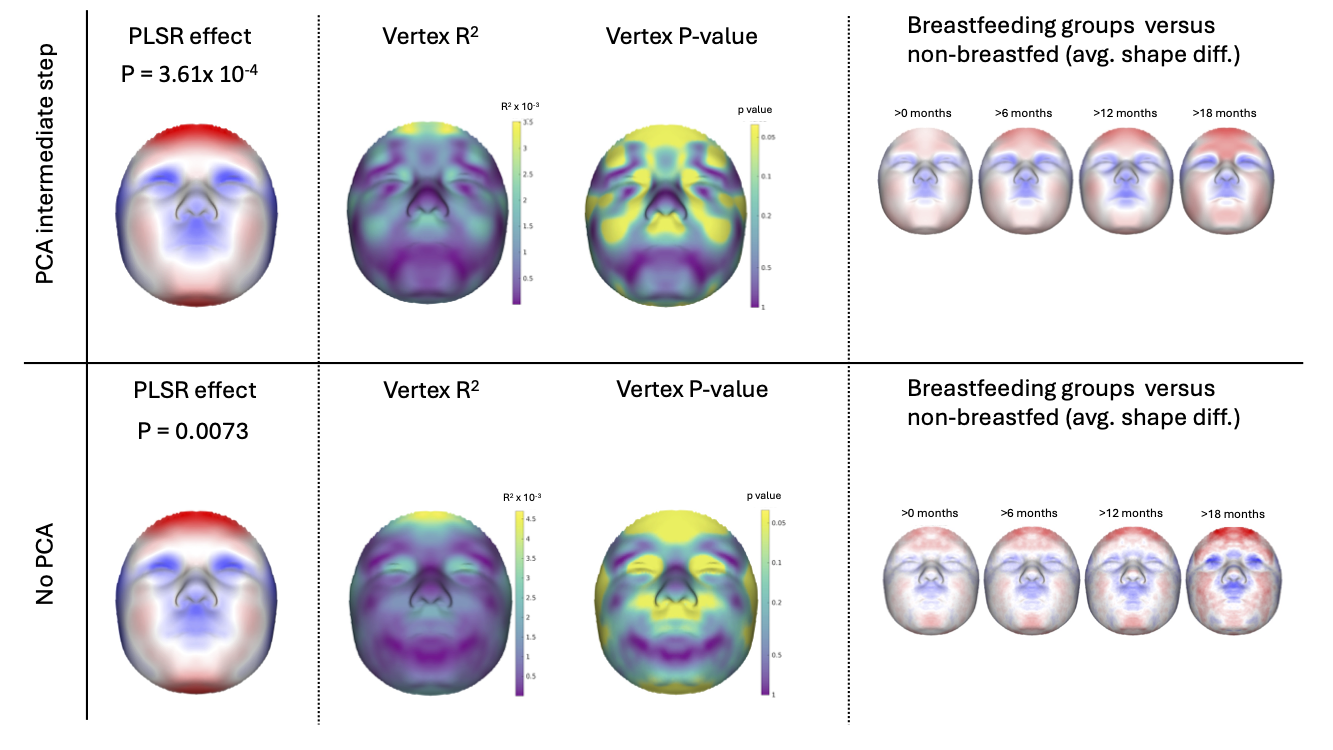

Supplement: S1 Fig — The top row shows the results as presented throughout the manuscript. To obtain these results, PCA was performed on facial shape and higher-order, noisy components were omitted. The bottom row shows the same semi-quantitative analyses without the intermediate PCA step, i.e., performed directly on the landmarks. In the first and last column, blue indicates that the breastfed group shows inward retrusion relative to the non-breastfed group, while red indicates outward protrusion. The vertex-wise variance explained by the semi-quantitative breastfeeding variable was estimated through PLSR and a corresponding P-value was obtained empirically through permutation testing. (TIFF) [file pone.0310538.s003.tiff]
